# Defense traits in the long‐lived Great Basin bristlecone pine and resistance to the native herbivore mountain pine beetle

**DOI:** 10.1111/nph.14191

**Published:** 2016-09-09

**Authors:** Barbara J. Bentz, Sharon M. Hood, E. Matthew Hansen, James C. Vandygriff, Karen E. Mock

**Affiliations:** ^1^ USDA Forest Service Rocky Mountain Research StationLoganUT84321USA; ^2^ USDA Forest Service Rocky Mountain Research StationMissoulaMT59808USA; ^3^ Department of Wildland Resources and Ecology CenterUtah State UniversityLoganUT84321USA

**Keywords:** climate change, *Dendroctonus ponderosae* (mountain pine beetle), exaptation, *Pinus balfouriana* (foxtail pine), *Pinus flexilis* (limber pine), *Pinus longaeva* (Great Basin bristlecone pine), tree longevity

## Abstract

- Mountain pine beetle (MPB,* Dendroctonus ponderosae*) is a significant mortality agent of *Pinus*, and climate‐driven range expansion is occurring. *Pinus* defenses in recently invaded areas, including high elevations, are predicted to be lower than in areas with longer term MPB presence. MPB was recently observed in high‐elevation forests of the Great Basin (GB) region, North America. Defense and susceptibility in two long‐lived species, GB bristlecone pine (*Pinus longaeva*) and foxtail pine (*P. balfouriana*), are unclear, although they are sympatric with a common MPB host, limber pine (*P. flexilis*).
- We surveyed stands with sympatric GB bristlecone–limber pine and foxtail–limber pine to determine relative MPB attack susceptibility and constitutive defenses.
- MPB‐caused mortality was extensive in limber, low in foxtail and absent in GB bristlecone pine. Defense traits, including constitutive monoterpenes, resin ducts and wood density, were higher in GB bristlecone and foxtail than in limber pine.
- GB bristlecone and foxtail pines have relatively high levels of constitutive defenses which make them less vulnerable to climate‐driven MPB range expansion relative to other high‐elevation pines. Long‐term selective herbivore pressure and exaptation of traits for tree longevity are potential explanations, highlighting the complexity of predicting plant–insect interactions under climate change.

## Introduction

A well‐documented consequence of recent climatic change is alteration in the geographic ranges of species (Lenoir *et al*., 2008; Mason *et al*., 2015), often causing complex, cascading, negative effects on biodiversity and community dynamics in newly invaded ecosystems (Forister *et al*., 2010). In many insects, variation in temperature‐dependent developmental rates, life cycle timing and long‐distance dispersal provide the capacity to track rapidly changing climatic conditions (Bale *et al*., 2002). In addition to appropriate thermal regimes, range expansion of herbivorous insects requires suitable host plant resources. Long‐term contact and evolutionary history between specific plant and herbivorous insect species are expected to increase plant defenses (Herms & Mattson, 1992), and the resource availability hypothesis (RAH) predicts that slow‐growing plants will invest heavily in defenses against herbivore attack and feeding because of the high cost of replacing tissue (Coley *et al*., 1985). Therefore, plants encountered by insects as a result of climate change‐driven range expansion may or may not be suitable hosts, depending on the level and form of defense.

When non‐native herbivores are introduced into novel plant systems, plants typically are naïve (i.e. have no previous contact) and therefore lack targeted defense traits, making them highly susceptible to herbivory (Desurmont *et al*., 2011). In ecosystems experiencing climate change‐induced expansion of native insect herbivores, however, evolutionary history between plant and insect can be more complicated, especially in long‐lived tree species. In temperate regions, post‐glacial range shifts of plants and their insect predators can become decoupled, resulting in non‐overlapping distributions despite long pre‐histories of contact (Hill *et al*., 2011). Plant species are expected to quickly lose expensive defense mechanisms in the absence of herbivore pressure in such situations. Long‐lived tree species, however, may retain evolutionary signals of past selection (Hamrick, 1979), even during periods of reduced herbivore pressure.

Disrupted evolutionary contact and subsequent loss of host plant defenses, in addition to plant naivety, are hypothesized factors driving the climate change‐induced invasion of the mountain pine beetle (MPB, *Dendroctonus ponderosae* Hopkins, Coleoptera: Curculionidae, Scolytinae) into *Pinus* habitats of Canada (Cudmore *et al*., 2010; Erbilgin *et al*., 2014). MPB is an irruptive herbivore native to *Pinus* ecosystems across western North America. The ranges of *Pinus* species have historically fluctuated, however, and currently extend further north and south than MPB's documented range (Wood, 1982). The current extent of *Pinus* in western Canada, for example, was a result of rapid post‐glacial expansion (Godbout *et al*., 2008, 2012) into areas which were generally too cold for MPB population success (Safranyik & Carroll, 2006). In the mid‐1990s, however, warming was associated with MPB population irruptions that caused mortality on c. 28 million ha in the western USA and Canada (USDA Forest Service, 2015; British Columbia, Ministry of Forests, Lands and Natural Resource Operations, 2012), including lodgepole pine (*Pinus contorta* Douglas) and jack pine (*P. banksiana* Lamb) ecosystems in Canada with no previous record of MPB exposure (Cudmore *et al*., 2010; Cullingham *et al*., 2011). Relative to lodgepole pine populations within the historical MPB distribution, lodgepole and jack pines in the newly invaded habitats have reduced defenses, including lower total concentrations and altered ratios of secondary compounds known to play important roles in MPB attraction to and success in *Pinus* hosts (Clark *et al*., 2014; Burke & Carroll, 2016).

Extensive MPB‐caused tree mortality has also recently occurred in high‐elevation *Pinus* ecosystems that include whitebark pine (*P. albicaulis* Engelm.) (Macfarlane *et al*., 2013) and limber pine (*P. flexilis* James) (Cleaver *et al*., 2015). Similar to expansion northward, recent MPB attack and reproductive success at high elevations are hypothesized to have resulted from warming temperatures (Weed *et al*., 2015; Bentz *et al*., 2016) and an absence of evolved tree defenses, presumably lost during post‐glacial pine species redistributions as unfavorable climatic conditions limited MPB success (Raffa *et al*., 2013). Redistribution of whitebark pine is known to have occurred following glaciation (Richardson *et al*., 2002), and an association between whitebark pine and MPB during this time period has been documented (Brunelle *et al*., 2008), although pre‐glacial association between MPB and *Pinus* is unknown. MPB was also documented infesting whitebark pines periodically throughout the 1900s, although the duration of population outbreaks and associated tree mortality was considerably less than that documented during the recent warming event (Perkins & Swetnam, 1996; Furniss & Renkin, 2003; Macfarlane *et al*., 2013). Extensive tree mortality in these high‐elevation *Pinus* ecosystems is troubling, as they are foundational species playing significant ecological roles and have relatively advanced age until reproduction (McCaughey & Tomback, 2001). Moreover, observed climate change‐induced mortality in whitebark and limber pines suggests that other high‐elevation *Pinus* could be highly susceptible to MPB. Great Basin (GB) bristlecone pine (*P. longaeva* Bailey) is a high‐elevation species occurring within the current geographic range of MPB, yet its vulnerability to MPB is unclear.

GB bristlecone pine is a Tertiary relic confined to cold, dry, high‐elevation sites in the GB region of the western USA. As with other *Pinus* species (Godbout *et al*., 2008; Potter *et al*., 2015), its distribution has fluctuated greatly during Pleistocene glaciation and Holocene warming (Wells, 1983). During the pleniglacial cool period (> 37 000 yr before present (bp)), GB bristlecone formed extensive low‐elevation forests, growing as low as 800 m below current distributions (Wells, 1983). Limber pine was a common associate of GB bristlecone pine and, during post‐glacial Holocene warming, both species retreated upward to the remote sky island mountain ranges they inhabit today (Thompson & Mead, 1982). GB bristlecone pine is known for its ability to attain great ages and survive adverse growing conditions (Schulman, 1958). It has the longest lifespan of any non‐clonal organism world‐wide, including other long‐lived *Pinus* species (Supporting Information Table S1). Recent aerial surveys in the GB region recorded MPB activity in high‐elevation areas of mixed GB bristlecone and limber pine (USDA Forest Service, 2015), although MPB‐caused GB bristlecone pine mortality remains unconfirmed (Gray *et al*., 2015).

Our goals were to determine whether GB bristlecone pine is a suitable host for MPB, and to assess its defense characteristics and level of resistance relative to other high‐elevation *Pinus* species in the region. If the ranges of GB bristlecone and MPB either historically never overlapped, or if historical contact was lost post‐glaciation, GB bristlecone would be predicted to have low defense levels and to be highly vulnerable to MPB attack, similar to that hypothesized for other recently invaded *Pinus* ecosystems (Cudmore *et al*., 2010; Raffa *et al*., 2013). Alternatively, the longevity of GB bristlecone pine could provide the capacity to retain previously evolved defense traits despite a lack of continuous contact with phloem feeders (Hamrick, 1979). Furthermore, resin‐related traits, originally selected for the minimization of wood decay caused by abiotic stressors and thereby conferring extreme longevity (LaMarche, 1969), could be an exaptation (i.e. traits evolved for one purpose that are co‐opted for their current use) to phloem feeders including MPB.

We conducted field surveys across a large portion of the spatial distribution of GB bristlecone pine to determine whether it was attacked by MPB during a recent period of elevated tree mortality that began following warming in the middle 1990s. We focused on areas in which GB bristlecone pine grows in mixed stands with limber pine, a known susceptible MPB host, and where MPB activity was recorded. We also surveyed for MPB attacks on foxtail pine (*P. balfouriana* Grev. and Balf.), a sister species to GB bristlecone pine that can be long lived and is also potentially experiencing climate change‐induced contact with MPB. Defense traits of GB bristlecone and foxtail were assessed, relative to co‐occurring limber pine, at multiple sites across the range of each species. We focused on defense traits shown to be important in bark beetle attack on conifers, including constitutive resin chemical profiles, resin duct characteristics and wood density.

## Materials and Methods

### Study system

MPB population levels can increase rapidly when mature hosts are abundant and environmental conditions are suitable for successful brood development and survival (Safranyik & Carroll, 2006; Bentz *et al*., 2014). MPBs feed in the phloem of living trees > c. 12 cm in diameter, typically killing the host in the process. In general, *Pinus* species invest heavily in both permanently expressed constitutive defenses and stimulated induced defenses in response to attack (Moreira *et al*., 2014; Hood & Sala, 2015). Constitutive beetle‐related defenses include chemical and structural features of the outer bark, phloem and xylem (Franceschi *et al*., 2005). For example, *Pinus* produce resin stored in an interconnected network of axial and radial ducts (Wu & Hu, 1997). Resin can physically impede and is toxic to attacking beetles, and trees with greater levels of constitutive and induced resin compounds are considered to be more highly defended (Seybold *et al*., 2006; Raffa, 2014).

Although *Pinus* species have evolved physical and chemical defense traits, MPB has evolved counter‐adaptations to tolerate and even benefit from low levels of host‐produced secondary resin toxins (Franceschi *et al*., 2005; Keeling & Bohlmann, 2006; Blomquist *et al*., 2010). Monoterpenes are the most common and well‐studied resin compounds in *Pinus*, and species within the genus tend to be qualitatively similar, but quantitatively variable (i.e. chemically similar or identical monoterpenes, but different total amounts and ratios), and ratios of compounds can vary geographically within and between species (Zavarin *et al*., 1993; Latta *et al*., 2000; Taft *et al*., 2015). Because specific monoterpenes can be ecologically important in the MPB–host relationship, the composition of monoterpenes for a particular species probably reflects the evolutionary history between the *Pinus* species and MPB (Huber *et al*., 2004).

MPB's range extends almost 30° in latitude from Baja California Norte, Mexico to northern British Columbia and western Alberta, Canada, and the majority of *Pinus* within this range are considered as hosts (Wood, 1982). GB bristlecone pine and its close relatives, foxtail pine and Rocky Mountain bristlecone pine (*P. aristata* Engelm.), make up the Balfourianae subsection of *Pinus* (Eckert & Hall, 2006). These three species have the longest lifespans of all *Pinus* (> 2400 yr) and are estimated to have existed in the western USA for > 40 million yr (Lanner, 2007). GB bristlecone pine grows on isolated mountain ranges in Utah, Nevada and southern California, and foxtail pine grows in two disjunct populations in northern and southern California. Low levels of recent MPB‐caused mortality have been documented for Rocky Mountain bristlecone pine (Klutsch *et al*., 2011) and foxtail pine (Maloney, 2011), but not GB bristlecone pine (Gray *et al*., 2015).

### Study locations, stand metrics and MPB‐caused tree mortality

**Figure 1:**
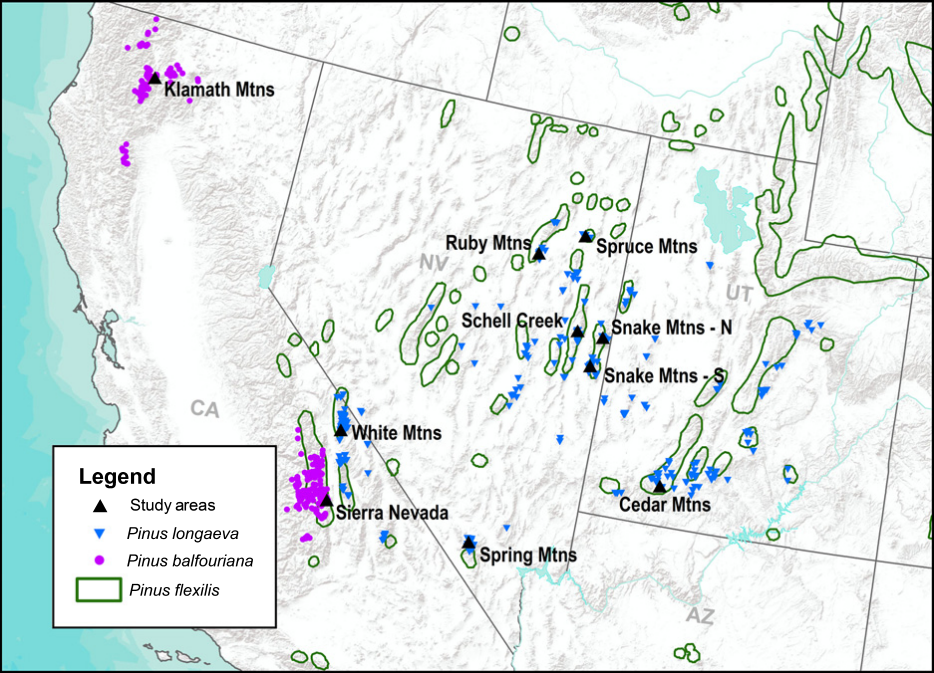
Distributions of Great Basin (GB) bristlecone (*Pinus longaeva*) and foxtail (*P. balfouriana*) pines based on published (Bailey, 1970; Kay & Oviatt, 1978; Matroguiseppe & Matroguiseppe, 1980; Oline *et al*., 2000; USDA Forest Service, 2005) and unpublished (J. Dunlap & C. Gray, pers. comm) sources. The distribution of limber pine (*P. flexilis*) reported by Little (1971) and the location of study sites are also shown.

To evaluate MPB preference for each species, MPB activity between 1997 and 2014 was determined from aerial surveys (USDA Forest Service, 2015) and overlain with known distributions of GB bristlecone, foxtail and limber pines. Foxtail and GB bristlecone distributions do not overlap, although both species overlap with limber pine's distribution. Study areas were chosen based on the presence of the target species (Fig. 1), MPB activity and practical road access.

Between June and September 2014, stands at ten mountain ranges across the ranges of GB bristlecone and foxtail pines were sampled and surveyed (Table S2; Fig. 1). Stands were surveyed for MPB activity and stand metrics within eight of the ranges, and trees were sampled for defense traits in six ranges. Surveys were not conducted in the White Mountains because of a lack of MPB activity, nor in the Klamath Mountains because of an active wildfire that prevented site access. A double sampling scheme of fixed‐radius plots and inter‐plot surveys was used. Fixed‐radius plots (0.05 ha) were established in a grid pattern, and used to characterize the number of trees attacked by MPB and stand composition and structure. Within each plot, all trees > 12.7 cm diameter at breast height (dbh) were identified to species, and dbh and status (i.e. live, MPB‐killed, other mortality) were recorded. All *Pinus* were examined for signs of MPB attack, including boring dust, pitch tubes and woodpecker foraging. Dead *Pinus* were further examined for characteristic J‐shaped egg galleries, larval mining and pupal chambers under the bark (Wood, 1982). The species identities of live and dead trees were determined based on multiple morphological characteristics (Table S3; Figs S1–S3). In addition to MPB, white pine blister rust caused by the invasive pathogen *Cronartium ribicola* J.C. Fisch is a significant threat to high‐elevation pines, and its expansion into the GB region is unclear. All five‐needle *Pinus* were therefore also visually assessed for white pine blister rust presence based on aeciospores and blisters. Dwarf mistletoe (*Arceuthobium* spp.) infection was assessed using a six‐class rating system (Hawksworth, 1977).

MPB‐caused tree mortality can be rare or clumpy on a landscape, resulting in high sample variance when using fixed‐radius plots. For a more robust sample of MPB‐caused tree mortality, a 100% survey of MPB‐attacked trees was also conducted between fixed‐radius plots, hereafter referred to as inter‐plot surveys. Only MPB‐attacked trees were recorded in these surveys, noting *Pinus* species and dbh, and stand metrics were inferred from the fixed‐radius plots. In the Schell Creek Range, two stands were sufficiently small such that a 100% survey of the entire stand was conducted.

### Defense metrics

#### Tree sampling

Equal numbers of limber and GB bristlecone were sampled in four geographically separated areas, three of which were also surveyed for MPB activity as described above (Table S2). Similarly, equal numbers of limber and foxtail pine were sampled in the Sierra Nevada. Limber pine does not occur in the Klamath Mountains, and only foxtail pine was sampled. At each area, 15 trees/species were sampled. Live trees (38–45 cm dbh, with no beetle attack or pathogen signs) of each species were selected. To quantify monoterpenes, a 2.5 × 2.5‐cm^2^ phloem plug was removed with a chisel from the north bole aspect of each tree. Phloem thickness (mm) was measured and tissue was placed in a vial, sealed, immediately placed on dry ice for transport and then stored at −40°C until processing. Two opposing 5‐mm‐diameter cores were taken from each tree as close to the ground as possible using a manual increment borer to obtain a cross‐section of wood containing annual rings from 2014 to the tree pith.

#### Constitutive phloem composition

Resin monoterpenes extracted from phloem tissue were analyzed using gas chromatography (GC) and the methods of Powell & Raffa (2011). Finely chopped tissue was placed into 2‐ml glass GC vials with 1 ml of hexane and agitated for 24 h. The hexane solution was filtered through glass wool into a second GC vial, the first vial was rinsed with 0.25 ml of hexane twice, and the filtered solution was added to the second vial for a final volume of 1.5 ml. We added 15 μl of 0.1% isobutylbenzene (IBB) to the vial as an internal standard, as IBB separated distinctly from the other compounds and has been used in several other studies of pine resin chemistry (Powell & Raffa, 2011). Phloem samples were dried for 1 wk at 25°C and weighed. Samples were analyzed using a Hewlett Packard (Santa Clara, CA, USA) 5890 Series II gas chromatograph with an Agilent Technologies (Santa Clara, CA, USA) Cyclodex‐B column (0.25 mm diameter × 30 m) with helium as the carrier gas. The initial oven temperature was 60°C for 15 min, with a 5°C rise min^−1^ to 160°C, and then held at 160°C for 10 min, for a total run time of 45 min. Eleven GC‐grade analytical standards ((+)‐α‐pinene, (−)‐α‐pinene, myrcene, (+)‐Δ‐3‐carene, (+)‐β‐pinene, (−)‐β‐pinene, R‐(+)‐limonene, γ‐terpinene, terpinolene, sabinene, 4‐allylanisole; Sigma‐Aldrich) of known *Pinus* monoterpenes were run for peak identification in the sample chromatograms. To identify additional peaks, subsamples of GB bristlecone and limber pines were analyzed using GC‐MS for a final total of 29 compounds, 24 of which were monoterpenes. The concentration of each compound (mg) was calculated by integrating the peak area, dividing by the IBB area and multiplying by the IBB density. Monoterpene concentrations were standardized by dry phloem sample mass (g) to calculate the absolute concentration (mg g^−1^ phloem) for each compound, and the concentrations of the 29 compounds were summed for the total concentration.

#### Wood density, resin ducts and tree age

One core from each tree was used to measure wood density (kg m^−3^). The sapwood and heartwood of each core, identified at the time of core collection, were separated and dried at 103–105°C for 72 h. Density was calculated using the water displacement method (Williamson & Wiemann, 2010). The second core was used to quantify annual tree growth and axial resin duct production. Cores were prepared using standard techniques (mounted and sanded until the cellular structure was visible through a binocular microscope); cores were scanned using an Epson platform scanner at 2400 dpi and ring widths were measured to the nearest 0.001 mm using CooRecorder v.7.7 (Cybis Elekronik and Data AB, Saltsjöbaden, Sweden). Resin ducts and core width were measured in ImageJ v.1.46r (National Institutes of Health, Bethesda, MD, USA) to the nearest 1 × 10^−7^ mm^2^ using the ellipse tool, and the calendar year in which each duct formed was assigned. Ducts for the most recent 15 yr, from 1999 to 2013, were measured. Using the traits defined in Hood & Sala (2015), the following resin duct metrics were calculated based on raw ring width values: (1) duct size (mean size of all ducts per annual ring; mm^2^); (2) duct production (total number of ducts per annual ring; no. yr^−1^); (3) total duct area (sum of duct size per annual ring; mm^2^ yr^−1^); (4) duct density (total number of ducts per annual ring divided by ring area; no. mm^2^ yr^−1^); and (5) relative duct area (total duct area divided by ring area × 100; % annual ring). Traits 1–3 are unstandardized, whereas traits 4 and 5 are standardized to ring area.

### Data analyses

Stand metrics before recent MPB‐caused tree mortality were estimated by recoding all MPB‐killed trees as live. For analyses of the influence of tree size on MPB attack preference, trees were assigned to a dbh class as: (1) < 25 cm; (2) ≥ 25 and < 35 cm; (3) ≥ 35 and < 45 cm; (4) ≥ 45 and < 55 cm; and (5) ≥ 55 cm. Differences in size among host tree species within a mountain range were tested using generalized linear mixed models (Glimmix, SAS 9.4; SAS Institute Inc., Cary, NC, USA). Stand within mountain range was specified as a random variable and the response distribution was specified as log‐normal. Pairwise differences between tree species were tested using Tukey's *post‐hoc* test (α = 0.05). The influence of tree size on MPB attack (1, attack; 0, no attack) was tested using Glimmix with a binomial response distribution, and mountain range and stand within range were specified as random effects. Size differences within host species among ranges were also tested. Differences in phloem thickness and resin duct traits among host species were tested similarly. Preliminary analysis indicated strong correlations between unstandardized resin duct traits and ring area (duct size: *r *=* *0.76, *P *<* *0.0001; duct production: *r *=* *0.88, *P* < 0.0001; total duct area: *r *=* *0.90, *P *<* *0.0001); therefore, ring area was used as a covariate in these models. Ring area and the unstandardized resin duct traits were log‐transformed to stabilize residuals. Because tree age can influence wood density, approximate tree age and age‐by‐species covariates were used when testing for species differences in heartwood and sapwood density. Correlations (corr, SAS 9.4) among growth, resin duct traits, phloem thickness, wood density and total constitutive compounds were also tested.

Mixed models were used to test for species differences in total resin composition, with mountain range specified as random and a log‐normal response distribution. Log‐normal model estimates were back‐transformed for the reporting of mean and standard error. As a result of non‐normally distributed data, treatment differences in individual compounds were tested using a Kruskal–Wallis non‐parametric test with Dunn's *post‐hoc* test for multiple comparisons (Elliott & Hynan, 2011). Non‐metric multidimensional scaling (NMDS) (vegan library, v.2.0‐10) in R v.3.0.1 (R Core Team; http://www.Rproject.org/) was used to visualize and test similarities in resin composition among species and mountain ranges. All detected compounds were included, with the Bray–Curtis dissimilarity index used as the multidimensional distance measure. Stress values were used as a measure of the goodness of fit for the final NMDS configuration. Stress values < 0.05 indicate that the ordination provides an excellent representation of the data with no prospect of misinterpretation, values < 0.1 indicate a good representation with little risk of false inferences, and < 0.2 is usable (Clarke, 1993). Non‐overlapping 95^th^ percentile confidence interval ellipses indicate populations that are statistically different (α = 0.05) (Oksanen, 2015).

## Results

### Stand surveys

The number of stands and plots surveyed varied as a result of differences among mountain ranges (Table S4). Across the ranges surveyed, the GB bristlecone proportion of pine varied from 76% in the Snake Mountains South to 17% in the Ruby Mountains (Table S4). Foxtail pine comprised 26% of the pine component in the one range surveyed with that species. All surveyed stands had some level of MPB‐caused *Pinus* mortality. Of the 1163 GB bristlecone pine surveyed in fixed plots, we found none killed by MPB (Table 1). By contrast, 268 of 1575 limber pine (17%) and one of 85 foxtail pine (1.2%) in fixed plots were attacked and killed by MPB. Moreover, in inter‐plot surveys, we found an additional 1395 limber, seven foxtail and nine lodgepole pine killed by MPB, but no MPB‐killed GB bristlecone pine (Table 1).

**Table 1:** Number and percentage of trees attacked and killed by mountain pine beetle (MPB) in fixed‐radius plots and 100% surveys in eight mountain ranges containing either a mix of limber and Great Basin (GB) bristlecone pine or a mix of limber and foxtail pine

Range	*Pinus* species	Fixed‐radius plots & 100% surveys			Inter‐plot surveys
		% Trees MPB‐killed	Trees MPB‐killed	Trees alive	Trees MPB‐killed
Cedar Mountains	Limber	25.8	8	23	29
	GB bristlecone	0	0	50	0
Ruby Mountains	Limber	34.4	31	59	149
	GB bristlecone	0	0	19	0
Schell Range	Limber	7.2	19	246	44
	GB bristlecone	0	0	171	0
Sierra Nevada	Limber	4.2	9	204	96
	Foxtail	1.2	1	84	7
	Lodgepole	10.8	4	33	9
Snake Mountains North	Limber	19.25	82	344	619
	GB bristlecone	0	0	332	0
Snake Mountains South	Limber	7.1	8	105	2
	GB bristlecone	0	0	351	0
Spring Mountains^a^	Limber	28.1	48	123	82
	GB bristlecone	0	0	87	0
	Ponderosa	66.7	1	2	0
Spruce Mountains	Limber	24.0	63	200	374
	GB bristlecone	0	0	121	0

Percentage trees MPB‐killed is the proportion of each *Pinus* species attacked and killed by MPB. Also shown are the number of trees attacked and killed by MPB in inter‐plot surveys (*c*. 60 m wide) between fixed‐radius plots.

a One ponderosa pine was killed by western pine beetle (*Dendroctonus brevicomis* LeConte).

GB bristlecone pines were larger (dbh) than limber pines at four sites and similar in size at three sites, and foxtail and lodgepole pines were both larger than limber pine (Table S4). Limber pines > 25 cm dbh were more likely to be attacked by MPB than limber pines < 25 cm dbh (*F*_4,1570_ = 16.26, *P *<* *0.001). However, there were no attack rate differences among trees in dbh classes > 25 cm (Table S5).

In the Spring Mountains, 19 dead GB bristlecone and three dead limber pines had severe dwarf mistletoe infection. Across all sites, 22 dead GB bristlecone pine showed evidence of foraging by wood borer species (i.e. Cerambycidae and Buprestidae) that typically infest trees killed or severely weakened by other factors, including drought (Table S6). Four dead GB bristlecone pines had at least one parent gallery of MPB, although there was no evidence of egg hatch, larval mining, pupal chambers or brood adult emergence. We found no signs of white pine blister rust infection on any five‐needle *Pinus* species.

**Figure 2:**
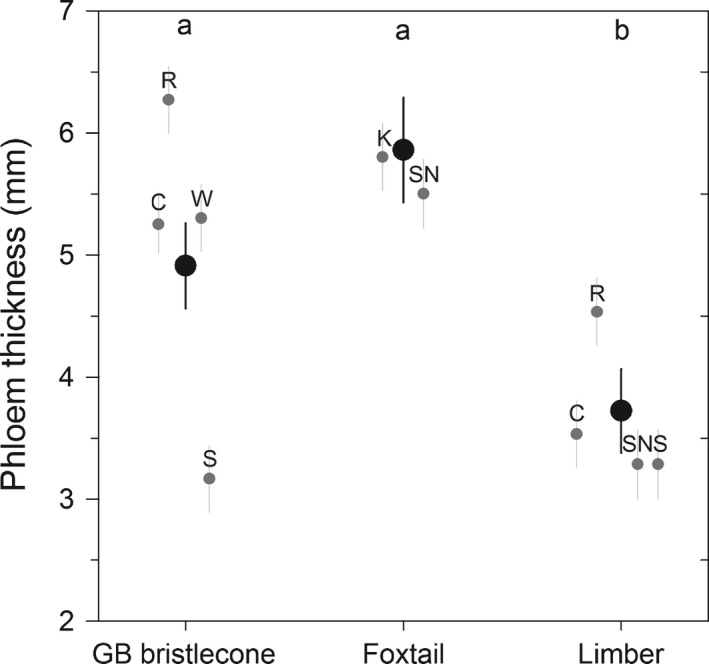
Mean phloem thickness (± SE) of Great Basin (GB) bristlecone, foxtail and limber pines. Black, species means; gray, mountain range means (C, Cedar; K, Klamath; R, Ruby; S, Spring; SN, Sierra Nevada; W, White; see Fig. 1; Supporting Information Table S1). Different letters at the top of the panel denote statistically significantly different species means (*P *<* *0.05). GB bristlecone pine phloem thickness was significantly thinner at the Spring Mountains than at the Cedar Mountains (*t*_142_ = 5.71, Adj. *P *<* *0.0001), Ruby Mountains (*t*_142_ = 7.95, Adj. *P *<* *0.0001) and White Mountains (*t*_142_ = −5.47, Adj. *P *<* *0.0001). No geographic differences were observed in phloem thickness among foxtail or limber pine populations.

Across all mountain ranges, limber pine had thinner phloem than GB bristlecone (*t*_144_ = 5.29, Adj. *P *<* *0.0001) and foxtail (*t*_144_ = 5.66, Adj. *P *<* *0.0001) pines (Fig. 2). Foxtail phloem tended to be slightly thicker than GB bristlecone pine phloem (*t*_144_ = −2.25, Adj. *P *=* *0.0663). GB bristlecone pine phloem thickness was significantly thinner at the Spring Mountains than all other mountain ranges in which it was sampled (Fig. 2). There was no relationship between phloem thickness and dbh for any species (*P *=* *0.1468), probably because only trees of 38–45 cm dbh were sampled. Sampled GB bristlecone pines were older (210 ± 27 yr) than foxtail (132 ± 20 yr) and limber (137 ± 18 yr) (*F*_2,145_ = 16.13, Adj. *P *<* *0.001) pines.

### Defense metrics

#### Constitutive phloem composition

**Figure 3:**
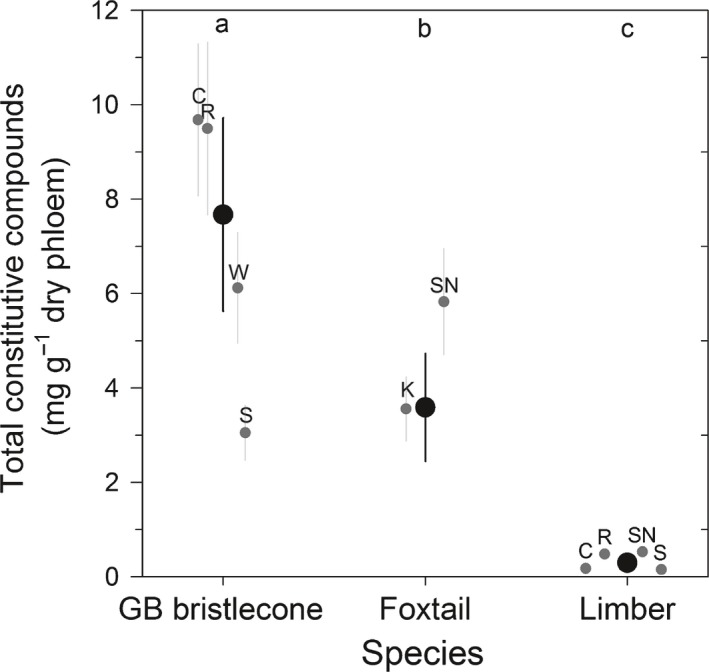
Total constitutive compound concentration (± SE) in phloem tissue of Great Basin (GB) bristlecone, foxtail and limber pines. Black, species means; gray, mountain range means (C, Cedar; K, Klamath; R, Ruby; S, Spring; SN, Sierra Nevada; W, White; see Fig. 1; Supporting Information Table S1). Different letters at the top of the panel denote significantly different species means (*P *<* *0.05). GB bristlecone sampled in the Spring Mountains had less total constitutive compounds than trees sampled in the Ruby and Cedar ranges (*t*_143_ = 4.18, Adj. *P *=* *0.0020 and *t*_143_ = 4.56, Adj. *P *=* *0.0005, respectively), and limber pine from the Sierra Nevada and Ruby ranges had greater total constitutive compounds than trees sampled from the Cedar and Spring ranges (Sierra Nevada vs Cedar *t*_143_ = −3.78, Adj. *P *=* *0.0084 and Sierra Nevada vs Spring *t*_143_ = 4.26, Adj. *P *<* *0.0001; Ruby vs Cedar *t*_143_ = −3.51, Adj. *P *=* *0.0204 and Ruby vs Spring *t*_143_ = 4.00, Adj. *P *=* *0.0039).

We focused on monoterpene composition in the constitutive phloem tissue of live, unattacked trees, although we detected additional compounds important in conifer–bark beetle interactions. These included the phenylpropene 4‐allylanisole and the oil eucalyptol (1,8‐cineole). We also observed two hydrocarbons and one unidentified compound (Table 2). Hereafter, we collectively refer to monoterpenes and other identified compounds as constitutive compounds. Across all mountain ranges, the total concentration of compounds differed among the three tree species (Table 2; Fig. 3; *F*_2,145_ = 257.57, Adj. *P *<* *0.001). GB bristlecone pine had double the concentration of total compounds found in foxtail pine (*t*_145_ = 2.77, Adj. *P *=* *0.0173), and more than eight times the concentration found in limber pine (*t*_145_ = 21.32, Adj. *P *<* *0.0001). The concentration of total compounds in foxtail pine was also greater than that in limber pine (*t*_145_ = 9.93, Adj. *P *<* *0.0001). Within‐species differences in total constitutive compounds varied by mountain range. In particular, GB bristlecone pine in the Spring Mountains had the lowest of all sampled sites for this species (Fig. 3). Total constitutive compounds were positively correlated with phloem thickness, and sapwood and heartwood density (*P *<* *0.0001) (Table S7).

**Table 2:** Percentage occurrence of phloem tissue constitutive compounds in Great Basin (GB) bristlecone, foxtail and limber pine

Compound	GB bristlecone	Foxtail	Limber	GB bristlecone	Foxtail	Limber	GB bristlecone	Foxtail	Limber
	*n *=* *65	*n *=* *30	*n *=* *58						
	% occurrence			Mean (SE), mg g^−1^			Mean (SE), %		
(−)‐α‐Pinene	100	100	91	2.2089 (0.220)^a^	2.6480 (0.395)^a^	0.0535 (0.009)^b^	27.68 (1.6)^a^	44.02 (4.0)^b^	11.22 (1.1)^c^
(+)‐α‐Pinene	100	100	100	4.6485 (0.377)^a^	2.4652 (0.403)^b^	0.1931 (0.031)^c^	55.97 (1.9)^a^	42.47 (4.6)^b^	38.75 (3.0)^b^
(−)‐β‐Pinene	98	93	98	0.2800 (0.032)^a^	0.1529 (0.023)^b^	0.0903 (0.024)^c^	3.52 (0.2)^a^	2.75 (0.3)^a^	16.74 (1.7)^b^
R‐(+)‐Limonene	98	100	45	0.0392 (0.003)^a^	0.0386 (0.004)^a^	0.0023 (0.000)^b^	0.53 (0.0)^a^	0.71 (0.0)^b^	0.64 (0.1)^a^
β‐Phellandrene	98	100	59	0.2263 (0.033)^a^	0.0430 (0.006)^b^	0.0184 (0.006)^c^	2.94 (0.4)^a^	0.78 (0.1)^b^	4.23 (1.1)^b^
Terpinolene	97	97	59	0.0737 (0.011)^a^	0.0637 (0.007)^a^	0.0090 (0.002)^b^	1.45 (0.3)^a^	1.20 (0.1)^a^	2.36 (0.4)^a^
(−)‐Camphene	94	90	17	0.0382 (0.003)^a^	0.0241 (0.004)^a^	0.0011 (0.000)^b^	0.47 (0.0)^a^	0.44 (0.1)^a^	0.15 (0.1)^b^
(+)‐β‐Pinene	94	97	45	0.0455 (0.004)^a^	0.0454 (0.006)^a^	0.0034 (0.001)^b^	0.65 (0.1)^ab^	0.90 (0.1)^a^	0.69 (0.1)^b^
(+)‐Camphene	92	100	12	0.0379 (0.003)^a^	0.0316 (0.003)^a^	0.0008 (0.000)^b^	0.50 (0.0)^a^	0.57 (0.0)^a^	0.22 (0.1)^b^
Myrcene	83	100	67	0.0312 (0.003)^a^	0.0338 (0.004)^a^	0.0244 (0.008)^b^	0.42 (0.1)^a^	0.62 (0.1)^b^	4.39 (0.8)^b^
Monoterpene 2	80	47	3	0.0174 (0.002)^a^	0.0076 (0.002)^b^	0.0001 (0.000)^c^	0.22 (0.0)^a^	0.11 (0.0)^b^	0.02 (0.0)^c^
Hydrocarbon 2	78	0	0	0.0161 (0.002)^a^	0^b^	0^b^	0.25 (0.0)^a^	0^b^	0^c^
Monoterpene 8	69	53	0	0.0084 (0.001)^a^	0.0056 (0.001)^a^	0^b^	0.10 (0.0)^a^	0.09 (0.0)^a^	0^b^
4‐Allylanisole	68	0	41	0.0252 (0.006)^a^	0^b^	0.0018 (0.000)^c^	0.30 (0.0)^a^	0^b^	0.41 (0.1)^a^
Monoterpene 1	66	37	2	0.0251 (0.003)^a^	0.0031 (0.001)^b^	Trace^c^	0.55 (0.1)^a^	0.07 (0.0)^b^	Trace^c^
Sabinene	66	47	45	0.0203 (0.005)^a^	0.0134 (0.004)^a,b^	0.0067 (0.002)^b^	0.36 (0.1)^a^	0.28 (0.1)^a^	1.67 (0.4)^a^
γ‐Terpinene	48	83	28	0.0076 (0.002)^a^	0.0125 (0.002)^a^	0.0071 (0.004)^a^	0.14 (0.0)^a^	0.26 (0.0)^b^	0.78 (0.3)^a^
(+)‐Δ‐Carene	32	53	43	0.1906 (0.088)^a^	0.1871 (0.050)^b^	0.0587 (0.017)^a,b^	3.63 (1.6)^a^	3.82 (0.9)^a^	14.26 (2.7)^a^
Monoterpene 6	23	27	0	0.0030 (0.001)^a^	0.0019 (0.001)^a^	0^b^	0.07 (0.0)^a^	0.03 (0.0)^a^	0^b^
Monoterpene 3	22	0	0	0.0021 (0.001)^a^	0^b^	0^b^	0.02 (0.0)^a^	0^b^	0^b^
Tricyclene	15	13	0	0.0012 (0.000)^a^	0.0017 (0.001)^a,b^	0^b^	0.01 (0.0)^a^	0.02 (0.0)^ab^	0^b^
Unknown 1	11	40	0	0.0045 (0.002)^a^	0.0359 (0.009)^b^	0^a^	0.06 (0.0)^a^	0.59 (0.2)^b^	0^a^
Monoterpene 4	9	10	9	0.0020 (0.001)^a^	0.0007 (0.000)^a^	0.0003 (0.000)^a^	0.05 (0.0)^a^	0.01 (0.0)^a^	0.04 (0.0)^a^
Eucalyptol (1,8‐cineole)	9	0	0	0.0011 (0.000)^a^	0^a^	0^a^	Trace^a^	0^a^	0^a^
Monoterpene 7	9	17	0	0.0006 (0.000)^a,b^	0.0023 (0.001)^b^	0^a^	0.01 (0.0)^a,b^	0.04 (0.0)^a^	0^b^
Monoterpene 5	5	20	0	0.0004 (0.002)^a^	0.0131 (0.042)^b^	0^a^	0.01 (0.0)^a^	0.20 (0.1)^b^	0^a^
β‐Ocimene	3	0	3	Trace	0	Trace	Trace	0	Trace
S‐(−)‐Limonene	0	3	9	0	Trace	Trace	0	Trace	Trace
Hydrocarbon 1	0	0	76	0^a^	0^a^	0.0135 (0.002)^b^	0^a^	0^a^	3.29 (0.4)^b^
Total				7.6712 (2.049)^a^	3.5862 (1.138)^b^	0.2951 (0.078)^c^			

Compounds are listed in decreasing commonness for GB bristlecone pine. Also shown are the mean (SE) of the absolute concentrations (mg g^−1^) and the mean (SE) percentage (%) of each compound by tree species. See Supporting Information Table S1 for sample locations. Differences among tree species in the concentrations of each compound are noted by different letters (α > 0.05).

Compounds also differed qualitatively among and within tree species. GB bristlecone and foxtail pine had several compounds, including tricyclene and four other unidentified monoterpenes (monoterpenes 5–8), which were either not found in limber pine or found in only a few trees (Table 2). Monoterpene 3, eucalyptol and hydrocarbon 2 only occurred in GB bristlecone pine. Both enantiomers of camphene were found in < 20% of limber pines, but > 90% of GB bristlecone and foxtail pines, and R‐(+)‐limonene was only found in 45% of limber, but more than 98% of GB bristlecone and foxtail pines. Hydrocarbon 1 only occurred in limber and 4‐allylanisole was found in GB bristlecone and limber, but not foxtail pine. The number of identified compounds was greatest in GB bristlecone pine, and the same compounds were present in all sampled GB bristlecone pine populations (Table 2). Given the higher levels of total compounds in GB bristlecone and foxtail relative to limber pine, it is not surprising that these species had greater absolute concentrations of the majority of compounds (Table 2). However, limber pine contained a proportionally greater amount of several compounds, including (−)‐β‐pinene, β‐phellandrene, terpinolene, myrcene and (+)‐Δ‐3‐carene (Table 2).

**Figure 4:**
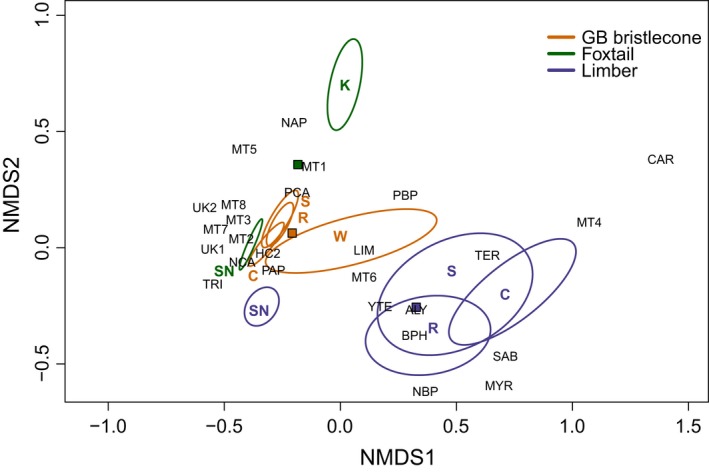
Non‐metric multidimensional scaling (NMDS) ordination of relative concentration (dimensions = 2, stress = 0.129) in Great Basin (GB) bristlecone, foxtail and limber pines. Squares, species means; ellipses, 95th percentile confidence interval (CI) of species mountain range location in ordination space, where: C, Cedar; K, Klamath; R, Ruby; S, Spring; SN, Sierra Nevada; W, White (see Fig. 1; Supporting Information Table S1). (−)‐α‐Pinene, NAP; (+)‐α‐pinene, PAP; (−)‐β‐pinene, NBP; R‐(+)‐limonene, LIM; β‐phellandrene, BPH; terpinolene, TER; (−)‐camphene, NCA; (+)‐β‐pinene, PBP; (+)‐camphene, PCA; myrcene, MYR; sabinene, SAB; γ‐terpinene, YTE; (+)‐Δ‐3‐carene, CAR; tricyclene, TRI; monoterpene 1, MT1; monoterpene 2, MT2; monoterpene 3, MT3; monoterpene 4, MT4; monoterpene 5, MT5; monoterpene 6, MT6; monoterpene 7, MT7; monoterpene 8, MT8; 4‐allylanisole, ALY; unknown 1, UK1; hydrocarbon 1, HC1; hydrocarbon 2, HC2.

The amount of α‐pinene (both enantiomers) was similar in GB bristlecone and foxtail pines, and comprised > 84% of total compounds compared with < 50% in limber pine (χ^2^ = 62.73, *P *<* *0.0001). Enantiomeric ratios of this compound differed, however, and GB bristlecone pine contained a proportionally greater amount of (+)‐α‐pinene relative to the two other species, and foxtail pine contained a proportionally greater amount of (−)‐α‐pinene relative to both GB bristlecone and limber pine (Table 2). Geographic differences within and among species were apparent for relative concentrations of compounds (Fig. 4). The two foxtail pine populations separated into distinct groups, White Mountain GB bristlecone pines were different from all other GB bristlecone populations, and Sierra Nevada limber pines were different from all other sampled limber populations (Fig. 4). Several compounds were responsible for within‐species differences. Eighty per cent of Sierra Nevada foxtail pines contained compound Unknown 1, but this compound was not present in any trees from the Klamath foxtail population, and monoterpene 6 was present in 50% of the Klamath samples, but only 10% of the Sierra Nevada samples (Table 2). Klamath foxtail pine also contained a greater amount of (−)‐α‐pinene than (+)‐α‐pinene, and the opposite was true in the Sierra Nevada population (Fig. S4). The Klamath foxtail population had a greater percentage of (+)‐Δ‐3‐carene relative to the Sierra Nevada population, Sierra Nevada limber pine had less of this compound relative to all other limber populations, and White Mountain GB bristlecones had a greater amount of (+)‐Δ‐3‐carene relative to other GB bristlecone pine populations (Fig. S4).

#### Wood density

**Figure 5:**
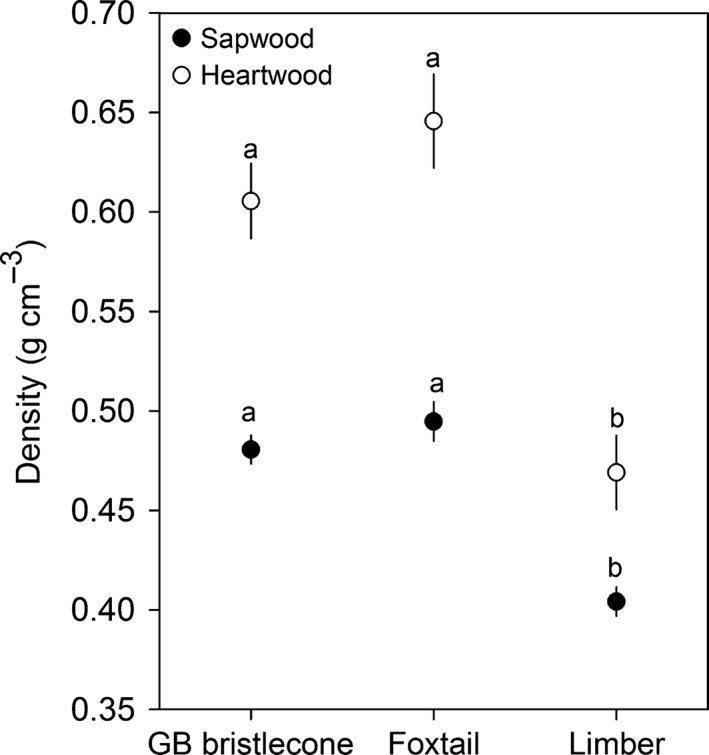
Mean (± SE) sapwood and heartwood density of Great Basin (GB) bristlecone, foxtail and limber pines after accounting for age effects. Different letters within xylem type denote statistically significantly different species means (*P *<* *0.05), using tree age as a covariate in the model to account for correlations between density and age.

Sapwood and heartwood densities differed by species (sapwood: *F*_2,141_ = 23.66, *P *<* *0.0001; heartwood: *F*_2,141_ = 12.21, *P *<* *0.0001). Limber pine sapwood and heartwood were less dense than those of GB bristlecone (sapwood: *t*_141_ = 9.77, Adj. *P* < 0.0001; heartwood: *t*_141_ = 9.84, Adj. *P *<* *0.0001) and foxtail (sapwood: *t*_141_ = 8.67, Adj. *P *<* *0.0001; heartwood: *t*_141_ = 8.25, Adj. *P *<* *0.0001) pines, but there were no differences in density between GB bristlecone and foxtail pines (sapwood: *t*_141_ = −1.24, Adj. *P *=* *0.4315; heartwood: *t*_141_ = −1.61, Adj. *P *=* *0.2455) (Fig. 5). Sapwood and heartwood densities declined with tree age (Table S8), and both were positively correlated with phloem thickness (*P *<* *0.0001) (Table S7).

#### Resin ducts

**Figure 6:**
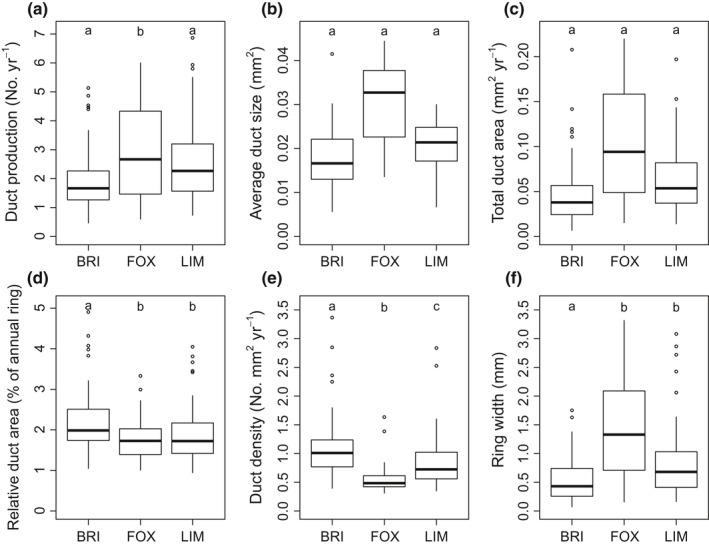
Average annual unstandardized (a–c) and standardized (d, e) resin duct characteristics and annual ring width (f) from the most recent 15 yr (1999–2013) of Great Basin bristlecone (BRI), foxtail (FOX) and limber (LIM) pines sampled across six mountain ranges in Utah, California and Nevada. Different letters indicate significant differences at *P *<* *0.05. Differences in unstandardized resin duct characteristics for (a–c) include ring area as a covariate in the model to account for correlations between resin ducts and growth. Boxes denote first and third quartiles, lines the median, whiskers the 1.5 interquartile range (IQR) and circles observations outside the 1.5 IQR.

For the unstandardized resin duct traits, only resin duct production differed among species, with greater production in foxtail than in GB bristlecone or limber pine (*F*_2,146_ = 5.47, *P *=* *0.0051; Fig. 6a). Duct size and total duct area did not differ among the three species (Fig. 6b,c). Annual ring area was a significant covariate in each of the unstandardized resin duct models (*P *<* *0.0001), reflecting the high correlations between growth and duct size and production. After accounting for annual ring width differences, GB bristlecone pine had higher relative duct area (% of annual ring area) and duct density (number per mm^2^ of xylem produced per year) than both foxtail and limber pines (Fig. 6d,e). Ring width was lowest in GB bristlecone pine (Fig. 6f). Phloem thickness and duct size and total duct area were positively correlated (*P *<* *0.0001) (Table S7). There was no relationship between any duct metric and total constitutive compounds.

## Discussion

### MPB attack preference

We found no evidence of MPB‐caused GB bristlecone pine mortality despite widespread proximal MPB activity. Although no GB bristlecone pines were killed by MPB in the mixed *Pinus* stands surveyed, MPB‐killed limber pines were common, and other *Pinus* species found infrequently in stands, including ponderosa (*P. ponderosa* Douglas) and lodgepole pine, were also attacked by MPB. Foxtail pine was killed, but at lower rates than sympatric limber and lodgepole pines. Our results are in contrast with previous studies conducted in mixed *Pinus* stands which showed relatively equal numbers of MPB‐killed lodgepole and whitebark pines (Bentz *et al*., 2015) and lodgepole and ponderosa pines (West *et al*., 2014). The absence of attacks on a *Pinus* species within MPB's range is particularly striking given that MPB successfully attacks and reproduces in several *Pinus* species exotic to North America (Furniss & Schenk, 1969), in addition to jack pine, a potentially naive *Pinus* outside MPB's post‐glacial range (Cullingham *et al*., 2011). Rather than being a highly susceptible and naive host for MPB, our results provide strong, quantitative corroboration of the observations of Gray *et al*. (2015) that MPB is not attracted to GB bristlecone pine.

MPB preference for large‐diameter trees with thick phloem is well documented (Safranyik & Carroll, 2006). In our study, MPB preference for limber rather than GB bristlecone or foxtail pine cannot be explained by tree size or phloem thickness. GB bristlecone and foxtail pines were either similar in size or larger than limber pine. Moreover, when standardized for tree diameter, both GB bristlecone and foxtail pines had thicker phloem than limber pine. Phloem thickness can be positively related to resin flow (Hood & Sala, 2015), however, and constitutive defenses, and resin chemistry in particular, probably explain why limber pine was preferred over GB bristlecone and foxtail pines.

### Defense metrics

GB bristlecone averaged almost eight times and foxtail almost four times greater total constitutive phloem concentration than in limber pine. Trees with greater concentrations of constitutive compounds are considered to be more resistant to bark beetles (Raffa, 2014). This result also aligns with the RAH which predicts that longer lived species in growth‐limiting environments should invest heavily in constitutive defenses because of a higher cost of replacing tissues from herbivory (Coley *et al*., 1985). Although differences in total concentrations among populations within a species can be significantly affected by abiotic stressors and environmental and genetic variation (Forrest, 1980; Ott *et al*., 2011), previous studies have found smaller and insignificant differences in total phloem constitutive compounds between *Pinus* species at a site (Raffa *et al*., 2013; West, 2013; Bentz *et al*., 2015). The large interspecific differences in constitutive compounds found at the same site, associated with reduced or no rates of attack on foxtail and GB bristlecone pines, suggest that total phloem compounds contributed to reduced MPB attacks. GB bristlecone pine had the thickest phloem and highest wood density, traits that were also positively correlated with total constitutive compounds. Although stress caused by severe dwarf mistletoe infection probably reduced GB bristlecone pine total constitutive compounds and phloem thickness at the Spring Mountain site (Nebeker *et al*., 1995), GB bristlecone total constitutive compounds were higher than in limber pine. Moreover, although dwarf mistletoe‐infected limber pines in the Spring Mountain stands were attacked, no mistletoe‐infected GB bristlecone pines were attacked by MPB, highlighting the effectiveness of GB bristlecone pine defenses.

Relationships between host monoterpenes and MPB host selection can be dose dependent and non‐linear, and a single compound or ratios of compounds can both facilitate and inhibit beetle response (Erbilgin *et al*., 2003). In addition to having greater total constitutive compounds, the majority of GB bristlecone and foxtail pine resin was α‐pinene, compared with < 50% in limber pine. Species with proportionally greater amounts of α‐pinene are considered to be easier to colonize and more attractive to foraging MPBs (Raffa *et al*., 2013; Burke & Carroll, 2016). Our result that the two *Pinus* species with the highest α‐pinene levels were also the least preferred by MPB suggests that host apparency and susceptibility to MPB are more complicated than the simple presence/absence of single compounds. In addition to facilitating attraction to host trees, α‐pinene is oxidized to verbenone, a ketone that repels beetles (Flechtmann *et al*., 1999; Lindgren & Miller, 2002), suggesting that there is an optimal α‐pinene level alone or in combination with other compounds for MPB attraction. Further, in addition to playing a role in beetle attraction, high concentrations of α‐pinene can inhibit beetle entry (Raffa, 2014).

Myrcene, a compound known to enhance attraction to MPB pheromones (Clark *et al*., 2014), was proportionally higher in the more susceptible limber pine than in GB bristlecone pine. Although relative proportions did not differ significantly among species, we found considerable variability in (+)‐Δ‐3‐carene concentrations among populations within a species. (+)‐Δ‐3‐Carene levels contribute to chemotypes in multiple *Pinus* species (Thoss *et al*., 2007; Taft *et al*., 2015), are strongly heritable (Baradat & Yazdani, 1988; Davis & Hofstetter, 2012) and play both negative and positive roles in tree attack and survival dynamics of MPB and its associates (Miller & Borden, 2000; Adams *et al*., 2011; Boone *et al*., 2013). The influence of (+)‐Δ‐3‐carene on MPB attraction and tree resistance deserves further investigation.

GB bristlecone and foxtail pine phloem tissue also contained several minor compounds either not found in limber pine or found in much lower tree frequencies and concentrations. These included a compound highly toxic to insects and an inhibitor of fungal activity (i.e. camphene; Mbata & Payton, 2013; Achotegui‐Castells *et al*., 2016), and compounds that may play a role in inhibiting bark beetle attacks (i.e. eucalyptol (1,8‐cineole) and (+)‐limonene; Schiebe *et al*., 2012). Although found in small concentrations, these compounds could play important roles in the MPB–tree interaction (McCormick *et al*., 2014), and should be investigated further for potential defense roles and management applications in tree protection against MPB attack.

In addition to greater resin concentrations, GB bristlecone and foxtail pines had denser sapwood and heartwood relative to limber pine. Wood density and bark density in tropical angiosperm species are positively correlated (Poorter *et al*., 2014), and denser bark could act as a physical deterrent to attacking beetles. Additional research is needed to determine whether this relationship extends to conifers. Wood density has been found to be positively correlated with tree survival, particularly in slow‐growing species, through numerous traits relating to water transport, nutrient acquisition, structural properties and pathogen resistance (Meinzer, 2003; Choat *et al*., 2008). We also found that wood density was positively correlated with phloem thickness.

GB bristlecone, foxtail and limber pines also differed in resin duct traits, which can increase *Pinus* survival from bark beetle attacks (Gaylord *et al*., 2013; Ferrenberg *et al*., 2014; Hood *et al*., 2015). Resin duct size and total resin duct area were strong predictors of ponderosa pine resin flow (Hood & Sala, 2015), suggesting that resin flow would not differ greatly among the three species studied here, as duct size and total duct area did not differ. Resin duct traits were strongly correlated with annual ring width, consistent with previous studies (Heres *et al*., 2014; Rodríguez‐García *et al*., 2014; Hood & Sala, 2015). Higher relative duct area and resin duct density, as found in GB bristlecone pine, have been related to increased resistance to MPB in other *Pinus* species (Ferrenberg *et al*., 2014; Gaylord *et al*., 2015; Hood *et al*., 2015). These traits, coupled with the narrower ring widths of GB bristlecone pine compared with foxtail and limber pines, suggest that GB bristlecone pine invests more strongly in resin duct defense than in growth compared with foxtail and limber pines. Although growth and unstandardized resin ducts are positively correlated, growth may be a more inconsistent measure of resistance. For example, Ferrenberg *et al*. (2014) found limber pine (but not lodgepole pine) with higher radial growth was more susceptible to MPB, but Hood *et al*. (2015) found no relationship between ponderosa pine growth and the resistance trait of resin flow. Resin flow has also been found to be both correlated (Westbrook *et al*., 2015) and uncorrelated (Lombardero *et al.,* 2000; Hood & Sala, 2015) with duct production (i.e. the number of ducts per ring), which was greatest in foxtail pine. We found that resin duct traits were positively correlated with phloem thickness, although there was no relationship between any resin duct metric and total constitutive compounds. Resin ducts, however, could play a role in induced *Pinus* responses, similar to that found in Norway spruce (Martin *et al*., 2002). In addition to the xylem ducts measured here, resin ducts are found in *Pinus* phloem. Although we did not measure phloem ducts, the observed positive relationship between phloem thickness and total constitutive compounds could be explained by the presence of larger numbers of resin ducts in thicker phloem. Research on species‐specific relationships between xylem and phloem resin duct traits and monoterpene synthesis is needed to understand relationships between resin duct anatomy and resin composition.

### Why are there species‐specific differences in defense traits?

The high investment in constitutive monoterpenes and structural traits found in GB bristlecone pine is consistent with predictions of the RAH. It is unclear, however, why the relatively long‐lived limber pine growing in the same stands had comparatively low investment in constitutive defenses, in addition to greater MPB susceptibility. As hypothesized for other *Pinus*, the comparatively high levels of constitutive defense traits may be the result of previous evolutionary pressure from phloem‐feeding herbivores (Raffa *et al*., 2013; Erbilgin *et al*., 2014). Extreme tree longevity could maintain these traits (Hamrick, 1979), regardless of disruption in contact with phloem feeders during past climate‐driven alterations in distribution. Alternatively, high resin production and wood density may have been originally adapted for the maintenance of extreme longevity, and were then co‐opted as exaptive defense traits against phloem feeders. Large amounts of resin and high wood density can limit wood decay and provide structural integrity to support living cambium, thereby also conferring longevity and survival in marginal habitats (LaMarche, 1969; Mourant *et al*., 2007; Brutovská *et al*., 2013). Evolution by exaptation has been shown to be important in the origin of new defense traits (Armbruster *et al*., 1997), just as adaptation of high‐elevation species to extreme climate stress may indirectly confer increased resistance to herbivores in a changing climate (Rasmann *et al*., 2014).

An additional explanation for species‐specific differences, meriting further exploration, is the potential for variability in induced defenses among and within *Pinus*. Induced defenses are an important tree response to MPB attack (Franceschi *et al*., 2005; Raffa, 2014), and the capacity for an induced reaction is unknown for the *Pinus* species included here. Plant defense theory and empirical evidence support the existence of trade‐offs between constitutive and induced defenses (Kempel *et al*., 2011; Moreira *et al*., 2014), suggesting that plants with high investment in constitutive defenses should invest little in induced defenses. We found that limber pine, a widely distributed species that grows across a wide elevational range, invests little in constitutive defenses, whereas GB bristlecone and foxtail pine, species restricted in their distributions, have high levels of constitutive compounds. The exploration of trade‐offs in induced and constitutive defenses among and within *Pinus* species will provide insights into evolutionary trade‐offs that confer long‐term vs short‐term protection against herbivores. Our results suggest that GB bristlecone pine is less vulnerable to climate‐driven range expansion of MPB than are other high‐elevation pines, and highlight the complexity of predicting insect–plant interaction outcomes in a changing climate.

## Author contributions

B.J.B., S.A.H., E.M.H., J.C.V. and K.E.M. planned and designed the research and contributed to manuscript writing. E.M.H., J.C.V. and B.J.B. performed field surveys and sampling. S.A.H. analyzed tree defense metrics and resin ducts. B.J.B. and S.A.H. analyzed the data and led the manuscript preparation.

## Supporting information

Please note: Wiley Blackwell are not responsible for the content or functionality of any Supporting Information supplied by the authors. Any queries (other than missing material) should be directed to the *New Phytologist* Central Office.

**Fig. S1** Photograph of Great Basin (GB) bristlecone and limber pine cones.
**Fig. S2** Photograph of foliage from dead Great Basin (GB) bristlecone pine.
**Fig. S3** Photograph of foliage from dead limber pine.
**Fig. S4** Relative proportion of compounds in constitutive phloem tissue of each tree species by mountain range.
**Table S1** Maximum recorded age for several *Pinus* species that occur in the western USA
**Table S2** Mountain range information for stand surveys and defense samples
**Table S3** Morphological characteristics used to distinguish among tree species
**Table S4** Stand and individual tree metrics within fixed‐radius and 100% surveyed stands
**Table S5** *Post‐hoc* tests for differences among diameter classes of trees attacked by mountain pine beetle (MPB)
**Table S6** Identification and voucher information of Cerambycid and Buprestid species associated with dead Great Basin (GB) bristlecone pine
**Table S7** Correlation coefficients (*r*) between tree growth and defense variables
**Table S8** Mixed model results for differences in heartwood and sapwood density among tree species
