# Supplementary material for: Defense traits in the long‐lived Great Basin bristlecone pine and resistance to the native herbivore mountain pine beetle
Source: New Phytol. 2016 Sep 9;213(2):611–24. doi: 10.1111/nph.14191 (PMC5213150; doi:10.1111/nph.14191)
Supplement: Supplementary file 1 — Fig. S1 Photograph of Great Basin (GB) bristlecone and limber pine cones. Fig. S2 Photograph of foliage from dead Great Basin (GB) bristlecone pine. Fig. S3 Photograph of foliage from dead limber pine. Fig. S4 Relative proportion of compounds in constitutive phloem tissue of each tree species by mountain range. Table S1 Maximum recorded age for several Pinus species that occur in the western USA Table S2 Mountain range information for stand surveys and defense samples Table S3 Morphological characteristics used to distinguish among tree species Table S4 Stand and individual tree metrics within fixed‐radius and 100% surveyed stands Table S5 Post‐hoc tests for differences among diameter classes of trees attacked by mountain pine beetle (MPB) Table S6 Identification and voucher information of Cerambycid and Buprestid species associated with dead Great Basin (GB) bristlecone pine Table S7 Correlation coefficients (r) between tree growth and defense variables Table S8 Mixed model results for differences in heartwood and sapwood density among tree species [file NPH-213-611-s001.pdf]

New Phytologist

***New Phytologist* Supporting Information**

Article title: **Defense traits in the long-lived Great Basin bristlecone pine and resistance to the native herbivore mountain pine beetle**

Authors: Barbara J. Bentz, Sharon A. Hood, E. Matthew Hansen, James C. Vandyriff and Karen E. Mock

Article acceptance date: 05 August 2016

The following Supporting Information is available for this article:

Fig. S1 Picture of GB bristlecone and limber pine cones.

Fig. S2 Picture of foliage from dead GB bristlecone pine.

Fig. S3 Picture of foliage from dead limber pine.

Fig. S4 Relative proportion of compounds in constitutive phloem tissue of each tree species by mountain range.

Table S1 Maximum recorded age for several *Pinus* species that occur in the western US.

Table S2 Mountain range information for stand surveys and defense samples.

Table S3 Morphological characteristics used to distinguish among tree species.

Table S4 Stand and individual tree metrics within fixed-radius and 100% surveyed stands.

Table S5 Post-hoc tests for differences among diameter classes of trees MPB-attacked.

Table S6 Identification and voucher information of Cerambycid and Buprestid species associated with dead GB bristlecone pine.

Table S7 Correlation coefficients (*r*) between tree growth and defense variables.

Table S8 Mixed model results for differences in heartwood and sapwood density among tree species.

New Phytologist

Fig. S1 Relatively weathered (top row) and fresh (bottom row) cones of GB bristlecone pine (left column) and limber pine (right column). The pocketknife is *c*. 7 cm long. Note the relatively smooth cone scales on limber pine and the armed barbs on GB bristlecone pine.

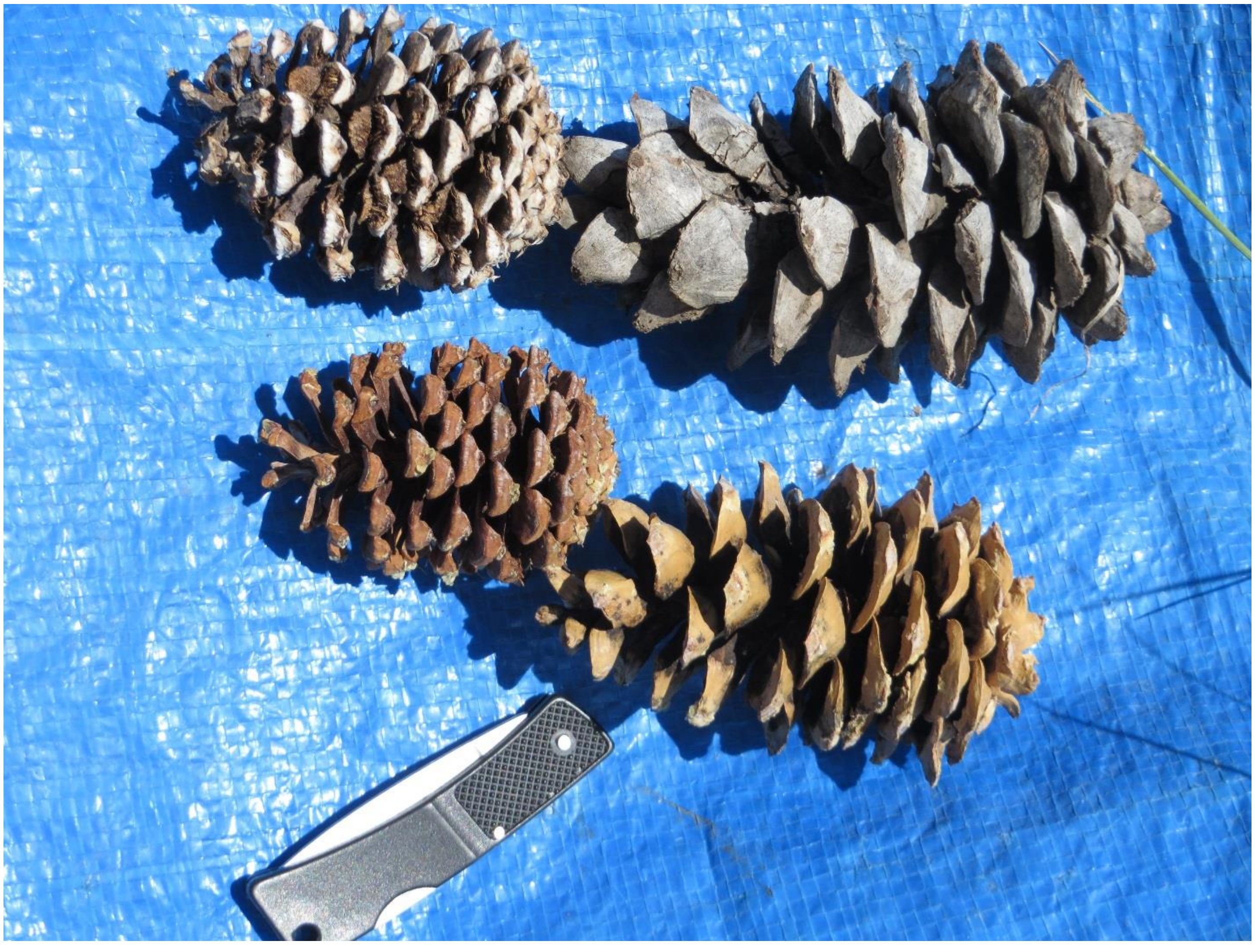

New Phytologist

Fig. S2 Foliage from a dead GB bristlecone pine retains the dense, bottlebrush texture indicative of the species. Relative to limber pine (Fig. S3), needles on dead GB bristlecone pine maintain their original shape and are more robust. Note the scaly texturing where the fascicles attach to the branch, a character that can remain well after all needles have fallen.

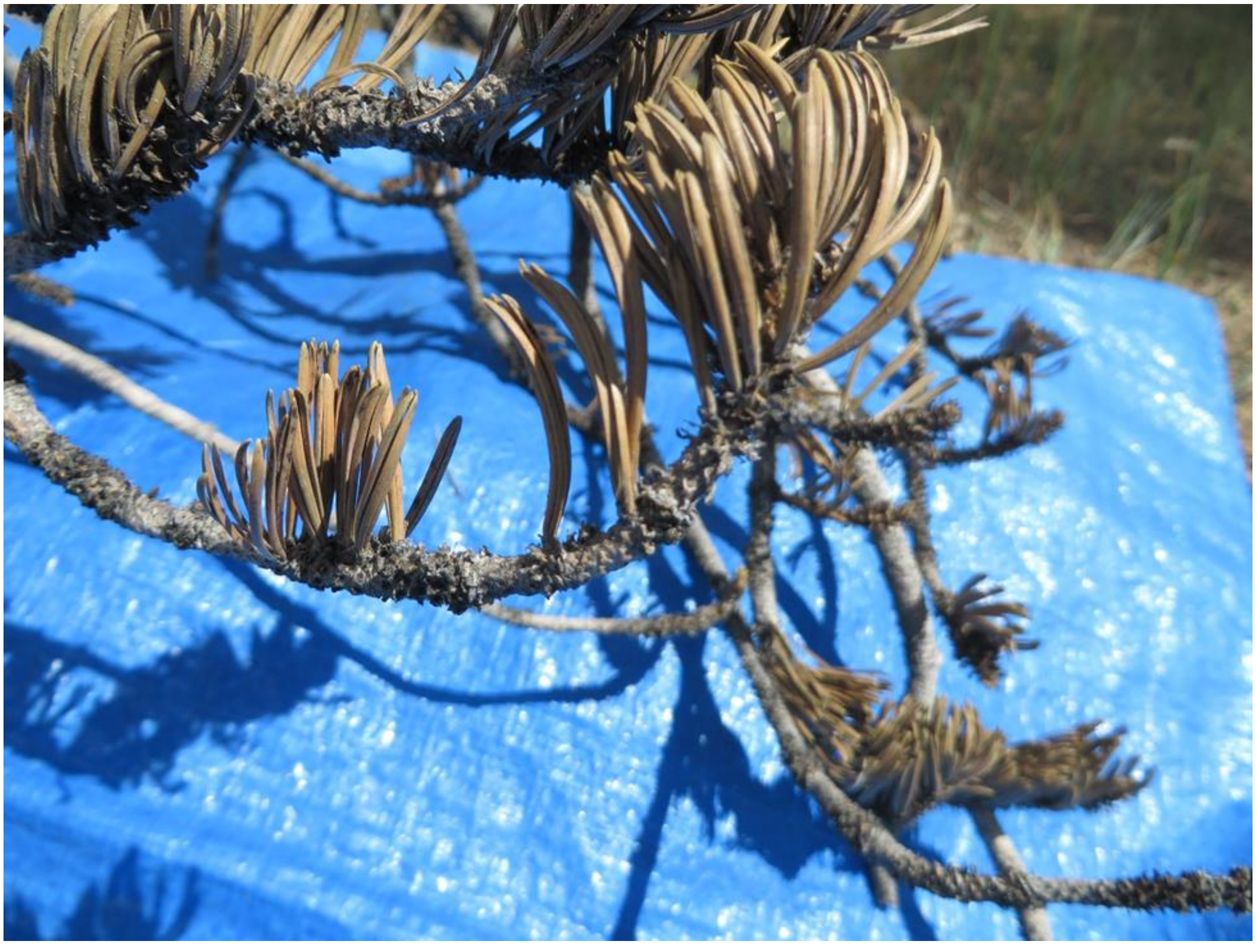

New Phytologist

Fig. S3 Foliage from a dead limber pine. Limber pine foliage is relatively less dense compared to that of GB bristlecone pine. Limber pine foliage is relatively slender or wispy and tends to curl increasingly with time since death. Note the relatively smooth texturing where the fascicle attaches to the branch.

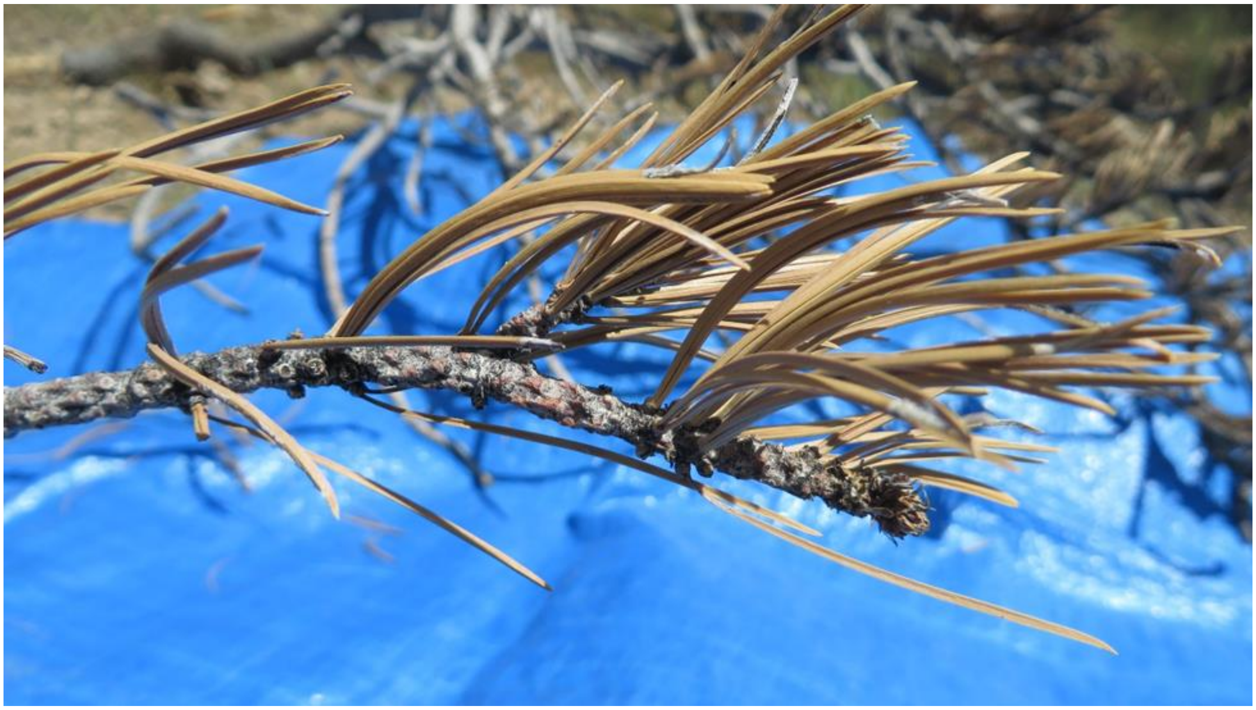

Fig. S4 Relative proportion of each compound (with presence > 1%) (± SE) in phloem constitutive tissue by tree species (GB bristlecone, foxtail and limber pine) and mountain range. (a) Cedar Mountains, (b) Spring Mountains, (c) Ruby Mountains, and (d) mountain ranges in California: White Mountains (WMnt), Sierra Nevada (SN) and Klamath Mountains (Klmth). See Fig. 1 and Table S2 for collection locations, and Table 2 for compound information. Note the different scale for α-pinene.

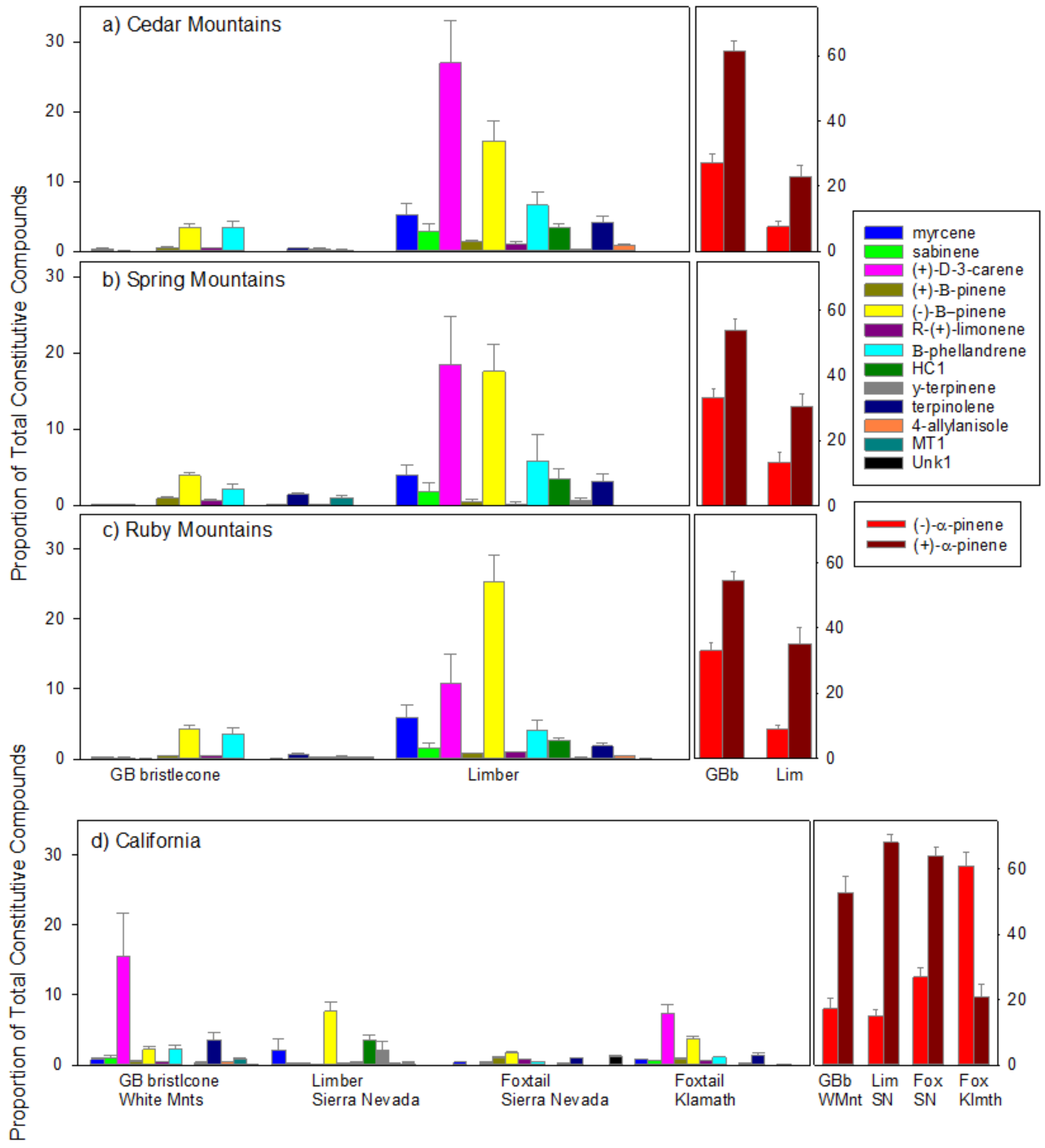

New Phytologist

Table S1 Maximum recorded age (yr) for several *Pinus* species that occur in the western United States.

Pinus species	Maximum recorded age (yr)
P. longaeva	4862–5062[1, 2]
P. aristata	2435[1, 2, 5]
P. balfouriana	2110–3400[1, 2, 6]
P. flexilis	1670–2030[1, 2, 3]
P. albicaulis	1267–1270[1, 4]
P. edulis	1101[2]
P. ponderosa	700–1016[7, 8]
P. monophylla	900[2]
P. lambertiana	400–500[9]
P. monticola	400[9]
P. contorta v. latifolia	400[9]

[1]Lanner, 2007; [2]http://www.rmtrr.org/oldlist.htm; [3]Johnson, 2004; [4]Perkins & Swetnam, 1996; [5]Brunstein *et al.*, 1992; [6]Ryerson, 1983; [7]Lanner, 1983; [8]http://www.monumentaltrees.com/en/usa/california/mariposacounty/12807_yosemitenationalpark/; [9]http://www.fs.fed.us/database/feis/plants/tree/

Table S2 Mountain ranges in Utah (UT), California (CA) and Nevada (NV), and associated pine species surveyed for mountain pine beetle activity. At some sites samples were also taken to characterize defensive traits, and at two sites (Klamath Mountains, White Mountains), only defensive trait samples were taken.

**Range**	***Pinus* species**	**Stand surveys**	**Defense sample**	**Elevation (m)**
Cedar Mountains, UT	GB bristlecone[1], Limber	Yes	Yes	2501–3042
Klamath Mountains, CA	Foxtail	No	Yes	1966–2233
Ruby Mountains, NV	GB bristlecone, Limber	Yes	Yes	2715–3106
Schell Creek Range, NV	GB bristlecone, Limber	Yes	No	3092–3191
Sierra Nevada, CA	Foxtail, Limber, Lodgepole	Yes	Yes	2863–3135
Snake Mountains North, NV	GB bristlecone, Limber	Yes	No	3052–3399
Snake Mountains South, NV	GB bristlecone, Limber	Yes	No	2989–3149
Spring Mountains, NV	GB bristlecone, Limber, Ponderosa	Yes	Yes	2764–2837
Spruce Mountain, NV	GB bristlecone, Limber	Yes	No	2930–3034
White Mountains, CA	GB bristlecone	No	Yes	3127–3171

[1]Great Basin bristlecone pine.

New Phytologist

Table S3 Characteristics used to distinguish between Great Basin (GB) bristlecone and limber pine mortality.

**Character**	**Description**
Cones	Persistent cones of limber pines are relatively large (8-15 cm long) with smooth cone scales and large, wingless seeds. GB bristlecone pine cones are relatively small (5–14 cm long) and the cone scales are armed with a pointed barb; seeds are small and winged (Fig. S1). Abundance of one cone type at the base of an open grown tree would strongly suggest the species.
Foliage	GB bristlecone pine have a dense bottlebrush foliar pattern and this pattern remains on trees that have recently died. GB bristlecone pine needles are relatively robust and tend to maintain their original form after tree death (Fig. S2). In contrast, limber pine needles are relatively slender, a character that becomes more conspicuous after tree death (Fig. S3). Additionally, because of their finer texture, limber pine needles are more prone to curl after death.
Fascicle attachment	On GB bristlecone pine, the attachment point of the fascicle and branch has a scaly texture (Fig. S2) which can persist well after needle-fall. In comparison, the attachment point of limber pine is relatively smooth (Fig. S3).
Epicormic branching	Epicormic branches on GB bristlecone pine are common. Relative to limber pine, a greater density of branches arise from a single location, and clumps of epicormic branches are consistent along the bole of GB bristlecone pine.
Bark	Although there can be considerable overlap in bark characteristics between the species, GB bristlecone pine has darker bark with deeper fissures and random bark scale shapes. The bark of dead GB bristlecone pine is difficult to penetrate with a sharp object. In contrast, limber pine bark is lighter colored with more regular blocky scales, and relatively soft and spongy on dead trees.

Table S4 Species composition, number of trees, basal area (BA), trees per ha (TPHA) and tree size (diameter at breast height, dbh) within fixed plots and 100% surveyed stands in mountain ranges prior to recent mountain pine beetle activity. Values were obtained by assuming trees recorded as killed by MPB were live prior to attack. Also shown are the number of stands and total number of fixed plots surveyed within each range. Stand surveys were not conducted at the White and Klamath mountains. Non-pine associates across the study stands included Douglas-fir (DF, *Pseudotsuga menziesii*), Englemann spruce (ES, *Picea englemanni*), quaking aspen (QA, *Populus tremuloides*), white fir (WF, *Abies concolor*), and subalpine fir (SAF, *Abies lasiocarpa*).

**Range**	**Species**	**No. trees (% of *Pinus*)**	**No. stands/plots**	**Mean dbh ± SE (cm)**	**BA m^2**	**TPHA**
Cedar Mountains	GB bristlecone	52 (62%)	1 / 7	43.54 ± 3.6[a]	31.6	148.6
	Limber	32 (38%)		29.82 ± 2.6[b]	8.6	91.4
	Non-pine[1]	40		20.96 ± 1.4	4.6	114.3
Ruby Mountains	GB bristlecone	19 (17%)	4 / 24	63.10 ± 7.9[a]	6.6	15.8
	Limber	92 (83%)		51.31 ± 2.6[b]	19.0	76.7
Schell Creek	GB bristlecone	171 (39%)	5[*] / 24	42.34 ± 1.7[a]	12.6	70.1
	Limber	265 (61%)		38.40 ± 1.1[a]	15.5	108.6
	Non-pine[2]	265		28.1 ± 0.8	8.1	120.8
Sierra Nevada	Foxtail	86 (26%)	3 / 44	57.27 ± 2.9[a]	12.1	39.1
	Limber	213 (63%)		43.13 ± 1.6[b]	18.5	96.8
	Lodgepole	37 (11%)		57.87 ± 4.0[a]	5.2	16.8
Snake Mountains-N	GB bristlecone	332 (44%)	5 / 75	40.38 ± 1.1[a]	14.5	89.7
	Limber	426 (56%)		39.43 ± 0.9[a]	17.1	115.1
	Non-pine[3]	233		27.95 ± 0.9	4.8	63.0
Snake Mountains-S	GB bristlecone	359 (76%)	4 / 45	45.52 ± 1.1[a]	30.7	159.6

	Limber	113 (24%)		40.05 ± 1.8[b]	7.7	50.2
	Non-pine[4]	52		30.13 ± 1.9	2.0	23.1
Spring Mountains	GB bristlecone	109 (39%)	2 / 20	28.41 ± 1.3[a]	8.0	109.0
	Limber	171 (60%)		27.70 ± 0.9[a]	12.1	171.0
	Ponderosa	3 (<1%)		62.65 ± 17.7[b]	1.1	3.0
	Non-pine[5]	256		24.85 ± 0.8	15.5	256.7
Spruce Mountain	GB bristlecone	121 (32%)	2 / 28	43.05 ± 2.0[a]	15.7	86.4
	Limber	263 (68%)		40.25 ± 1.1[a]	28.7	187.9
	Non-pine[6]	1		20.3	0.1	0.7

[*]Two stands were small enough for a 100% survey of all trees.

[#]Within a mountain range, species with different letters denote differences in dbh among pine species ($P < 0.05$).

[1]DF, ES, SAF; [2]ES, QA; [3]DF, ES, QA; [4]DF, ES, QA, WF; [5]QA, WF; [6]WF.

Table S5 Tukey-Kramer post-hoc tests for differences among diameter (dbh) classes in limber pine that were attacked by mountain pine beetle. Dbh was categorized into 5 classes: (1) < 25 cm, (2) >= 25 and < 35 cm, (3) >= 35 and < 45 cm, (4) >= 45 and <55 cm, and (5) >= 55 cm.

dbh class	dbh class	Estimate	Standard Error	DF	t Value	Pr > |t|	Adj P
**1**	**2**	-2.3265	0.3365	1570	-6.91	<.0001	<.0001
**1**	**3**	-2.4215	0.3398	1570	-7.13	<.0001	<.0001
**1**	**4**	-2.6482	0.3524	1570	-7.51	<.0001	<.0001
**1**	**5**	-2.7057	0.3525	1570	-7.68	<.0001	<.0001
**2**	**3**	-0.09507	0.2103	1570	-0.45	0.6512	0.9914
**2**	**4**	-0.3217	0.2245	1570	-1.43	0.1520	0.6061
**2**	**5**	-0.3792	0.2226	1570	-1.70	0.0887	0.4322
**3**	**4**	-0.2267	0.2240	1570	-1.01	0.3117	0.8500
**3**	**5**	-0.2841	0.2210	1570	-1.29	0.1988	0.7003
**4**	**5**	-0.05746	0.2270	1570	-0.25	0.8002	0.9991

Table S6 Wood borer (Coleptera: Cerambycidae, Burprestidae) species associated with dead Great Basin (GB) bristlecone pine in Nevada (NV) and California (CA). The three species are new records for association with GB bristlecone pine.

Species	Taxonomic Identification	Collection Location	Voucher location
Tetropium parallelum Casey	Jim LeBonte	Schell Creek Range, NV	Jim LeBonte, Oregon Department of Agriculture, Salem, OR
Chrysobothris falli Van Dyke	Rick Westcott	White Mountains, CA	Oregon State University, Corvallis, OR
C. monticola Fall	Rick Westcott	White Mountains, CA	Oregon State University, Corvallis, OR

Table S7 Correlation coefficients (*r*) between tree growth and defense variables. (*n* = 151). Significant correlations indicated by: **, *P*<0.01; ***, *P*<0.0001.

	Duct size	Total duct area	Relative duct area	Duct density	Ring width	Phloem thickness	Sapwood density	Heartwood density	Total constitutive compounds
Duct production	**0.72*****	**0.94*****	-0.08	**-0.32*****	**0.86*****	**0.26****	**0.24****	0.11	-0.04
Duct size		**0.85*****	-0.15	**-0.53*****	**0.78*****	**0.38*****	**0.32*****	**0.29****	0.04
Total duct area			-0.10	**-0.42*****	**0.90*****	**0.30*****	**0.33*****	**0.26****	-0.01
Relative duct area				**0.84*****	**-0.35*****	-0.11	-0.04	0.01	0.12
Duct density					**-0.53*****	**0.30****	-0.12	-0.08	0.03
Ring width						**0.30****	**0.32*****	**0.27****	-0.05
Phloem thickness							**0.44*****	**0.33*****	**0.59*****
Sapwood density								**0.79*****	**0.40*****
Heartwood Density									**0.39*****

Table S8 Mixed model results testing for differences in heartwood and sapwood density among three tree species, GB bristlecone, foxtail and limber pine. Estimates of tree age based on tree rings was included as a covariate.

Heartwood Density				
Effect	Num DF	Den DF	F Value	Pr > F
Tree species	2	141	12.21	<.0001
Age	1	141	8.91	0.0033
Age*tree species	2	141	1.53	0.2202
**Sapwood density**				
Effect	Num DF	Den DF	F Value	Pr > F
Tree species	2	141	23.66	<.0001
Age	1	141	16.91	<.0001
Age*tree species	2	141	2.76	0.0666

References

Brunstein FC, Yamaguchi DK. 1992. The oldest known Rocky Mountain bristlecone pines (*Pinus aristata* Engelm.). *Arctic and Alpine Research* **24**: 253–256.

Johnson CG. 2004. Alpine and subalpine vegetation of the Wallowa, Seven Devils and Blue Mountains. *USDA Forest Service, Pacific Northwest Region* (R6-NR-ECOL-TP-03-04), Portland, OR, USA.

Lanner RM. 1983. *Trees of the Great Basin: a natural history*. Reno, NV, USA: University of Nevada Press.

Lanner RM. 2007. *The bristlecone book: a natural history of the World's oldest trees.* Missoula, MT, USA: Mountain Press.

Perkins DL, Swetnam TW. 1996. A dendroecological assessment of whitebark pine in the Sawtooth-Salmon River region, Idaho. *Canadian Journal of Forest Research* **26**: 2123–2133.

Ryerson AD. 1983. *Population structure of* Pinus balfouriana *Grev. & Balf. along the margins of its distribution area in the Sierra and Klamath regions of California*. MS thesis, Sacramento State University, Sacramento, CA, USA.
